# Negative Index Metamaterial Lens for Subwavelength Microwave Detection

**DOI:** 10.3390/s21144782

**Published:** 2021-07-13

**Authors:** Srijan Datta, Saptarshi Mukherjee, Xiaodong Shi, Mahmood Haq, Yiming Deng, Lalita Udpa, Edward Rothwell

**Affiliations:** 1 Department of Electrical and Computer Engineering, Michigan State University, East Lansing, MI 48824, USA; shixiaod@msu.edu (X.S.); haqmahmo@egr.msu.edu (M.H.); dengyimi@egr.msu.edu (Y.D.); udpal@egr.msu.edu (L.U.); rothwell@egr.msu.edu (E.R.); 2 Lawrence Livermore National Laboratory, Livermore, CA 94550, USA; mukherjee5@llnl.gov

**Keywords:** metamaterial, lenses, refractive index, microwave sensors, nondestructive testing

## Abstract

Metamaterials are engineered periodic structures designed to have unique properties not encountered in naturally occurring materials. One such unusual property of metamaterials is the ability to exhibit negative refractive index over a prescribed range of frequencies. A lens made of negative refractive index metamaterials can achieve resolution beyond the diffraction limit. This paper presents the design of a metamaterial lens and its use in far-field microwave imaging for subwavelength defect detection in nondestructive evaluation (NDE). Theoretical formulation and numerical studies of the metamaterial lens design are presented followed by experimental demonstration and characterization of metamaterial behavior. Finally, a microwave homodyne receiver-based system is used in conjunction with the metamaterial lens to develop a far-field microwave NDE sensor system. A subwavelength focal spot of size 0.82λ was obtained. The system is shown to be sensitive to a defect of size 0.17λ × 0.06λ in a Teflon sample. Consecutive positions of the defect with a separation of 0.23λ was resolvable using the proposed system.

## 1. Introduction

In 1968, V. Veselago theoretically introduced the electrodynamics of materials having simultaneous negative values of permittivity ϵ and permeability μ [1]. He showed that such materials will exhibit unusual properties such as negative refraction, reversal of Doppler shift and backward Cherenkov radiation. The electric field, magnetic field, and wave vector of a plane wave form a left-handed triplet in such a medium, instead of the conventional right-handed one. The word “metamaterial” was coined for such materials, alluding to their unusual properties, not generally encountered in nature. The first left-handed metamaterial (LHM) structure was realized by Smith et al., in their seminal paper of 2000, where they showed that an alternating periodic array of split-ring resonators (SRRs), and thin wires can produce an effective medium having a negative refractive index in the microwave regime [2]. Extensive research demonstrating and characterizing the left-handed behavior of such structures followed [3,4,5]. Early on, negative refractive index structures were a controversial topic, and their existence was disputed by researchers [6,7]. However, over the past two decades, there has been significant evidence that certain periodic structures can indeed have an effective negative refractive index over a limited range of frequencies [8,9,10]. Such periodic structures, even though inhomogeneous, can behave as a homogeneous medium in response to electromagnetic (EM) waves with appropriately long wavelength. The homogenized negative index behavior of inhomogeneous metamaterial structures has been described by an effective negative ϵ and μ of the periodic arrays [11].

**Figure 1:**
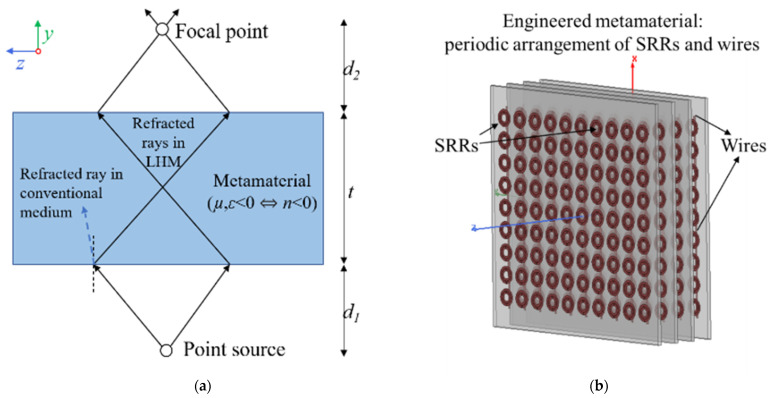
(**a**) Ray diagram showing reversal of Snell’s law in a metamaterial medium. For a conventional medium, the diverging beams from a point source will not come into focus. (**b**) Printed circuit board (PCB) implementation of a metamaterial consisting of alternating periodic arrangement of SRRs and wires. The structure will exhibit an effective negative refractive index over a range of frequencies under specific incident wave polarization.

Metamaterials have inspired many novel applications based on their negative refractive index. One of the most ingenious applications of LHM structures was put forward by J.B. Pendry, where he showed that a negative refractive index material can act as a “super lens”, capable of achieving subwavelength focusing in the far field by restoring the amplitude of evanescent wave components [12]. The highest resolution that can be obtained using a conventional lens in the far field is limited by the operating wavelength, due to the physics of diffraction. The breaking of this diffraction limit using point source focusing (Figure 1a) and evanescent wave amplification of a LHM lens has been one of the significant driving factors for metamaterial research. Various metamaterial designs, operating from radio to optical frequencies, have been developed and shown to achieve subwavelength focusing [13,14,15,16,17].

This paper reports the design of a metamaterial lens and its experimental implementation for far-field microwave detection of subwavelength defects. Far-field microwave NDE offers the advantage of rapid scan times, but is constrained by the diffraction limit from detecting smaller subwavelength defects [18]. A single SRR coupled with a transmission line behaves as a LC tank circuit, whose resonant frequency can be changed in the presence of a load. Although extensive research on such metamaterial-inspired near-field sensors have been described in literature, they do not offer the advantages of far-field systems [19,20,21,22]. While numerical studies of LHMs as lenses in the far field have been undertaken [23,24,25,26], the practical feasibility of their use has not been widely demonstrated. One study of far-field microwave imaging is reported by Shrieber et al., who present subwavelength defect detection in fiberglass composites [27]. An LHM lens-based microwave hyperthermia scheme for treatment of tumors is proposed in [28]. The metamaterial lens concept has been extended to ultrasonics as well [29], with various studies demonstrating subwavelength imaging using acoustic LHM lenses being reported [30,31,32]. The authors of the present paper recently reported a numerical study on enhancement of far-field microwave time-reversal imaging resolution using a homogenized model of a metamaterial lens [33]. Preliminary studies on the physical design of a metamaterial lens were presented by the authors in [34]. The present contribution focuses on detailed numerical and experimental characterization of the lens design and its feasibility for far-field subwavelength defect detection. Existing literature on using LHMs in the far field employs electromagnetic windowing techniques to realize subwavelength focusing or defect detection. In this paper, a homodyne receiver-based architecture is proposed to be used in conjunction with the metamaterial lens for far-field measurements. The high SNR associated with such synchronous detection allows the lens to be characterized in free space and, hence, provides a system that can be used in the field under practical conditions.

The theory governing the working principles of metamaterials is briefly discussed in the Section 2. The Section 3 provides a numerical study and EM parameter retrieval of the lens design using the commercial software HFSS. The Section 4 presents the experimental characterization of the metamaterial lens and its implementation for detection of subwavelength defects using the homodyne system.

## 2. Theory

A periodic array of conducting elements can act as an effective medium for EM scattering when the wavelength is much longer than the element dimensions, i.e.,

(1) a≪λ

where a is the dimension of a unit cell of the periodic array, and λ is the operating wavelength in the effective medium. The EM response of an effective medium is determined by the configuration of the unit cell and can be characterized by an effective relative permeability $\mu_{eff}$ and effective relative permittivity $\epsilon_{eff}$. Metamaterials with simultaneous negative $\mu_{eff}$ and $\epsilon_{eff}$ over a specific range of frequencies are termed as “double negative” metamaterials. One typical example of a unit cell comprises two distinct structures: an SRR element with dominant magnetic response and a thin wire element with dominant electric response.

A periodic array of the SRR elements can exhibit an effective magnetic behavior, similar to that of magnetic plasmas, in the microwave regime [35]. Under excitation by an external magnetic field parallel to the axis of the SRRs, the array behaves as a bulk medium having an effective relative permeability given by

(2) $$\mu_{eff}\left(\omega \right)=1-\frac{F\omega^{2}}{\omega^{2}-\omega_{0}^{2}+j\omega \Gamma }$$

where *F* is the fractional volume of the unit cell occupied by the rings, Γ is the dissipation factor, $\omega_{0}$ is the resonant frequency of the SRRs, and ω is the frequency of the excitation field. Equation (2) shows that the real part of $\mu_{eff}$ is negative for frequencies ω greater than resonant frequency $\omega_{0}$ and less than magnetic plasma frequency $\omega_{mp}$ given by $\omega_{mp}=\omega_{0}/\sqrt{1-F}$. Propagating wave modes are prohibited in this frequency band due to negative $\mu_{eff}$ of the SRR medium.

A periodic array of the thin metallic wire elements, under the influence of a time-varying electric field, can mimic an electric plasma at microwave frequencies [36]. The effective relative permittivity of such an array in the presence of an external electric field parallel to the wires is given by

(3) $$\epsilon_{eff}\left(\omega \right)=1-\frac{\omega_{ep}^{2}}{\omega^{2}}$$

where $\omega_{ep}$ is the electric plasma frequency, and ω is the frequency of the excitation field. Equation (3) shows that the real part of $\epsilon_{eff}$ has negative values for frequencies $\omega <\omega_{ep}$. This causes the wire medium to prohibit propagating modes in that frequency regime.

Combining both the SRR and wire elements in a periodic array gives rise to a metamaterial medium having simultaneous negative $\mu_{eff}$ and $\epsilon_{eff}$ over a certain range of frequencies. Assuming that there is no direct interaction between the SRR and wire media, the refractive index *n* of the resulting metamaterial is given through

(4) $$n^{2}=\mu_{eff}\left(\omega \right)\epsilon_{eff}\left(\omega \right)$$

The negative square root in the calculation of *n* is chosen in (4) when both $\mu_{eff}\left(\omega \right)$ and $\epsilon_{eff}\left(\omega \right)$ are negative to account for propagation of left-handed waves in a metamaterial [37]. A left-handed transmission band occurs within the previously overlapping forbidden bands of negative $\mu_{eff}$ and negative $\epsilon_{eff}$. The combined array behaves as a medium having an effective negative refractive index in this transmission band, and the transmission peak is referred to as a left-handed peak. Such a negative index medium not only focuses propagating waves but also enhances the evanescent wave component of the angular spectrum of the incident field, which contains high-resolution information. Subwavelength focusing beyond the diffraction limit is, thus, made possible by using a negative index metamaterial lens. As shown in Figure 1a, for an ideal lossless LHM lens of thickness *t* and refractive index *n* = −1, a diverging beam from a point source at a distance *d*_1_ from the lens focuses first inside the lens and, then, outside the lens at a distance *d*_2_ given by [12]

(5) $$d_{2}=t-d_{1}$$

Although the presence of losses associated with fabricated LHMs causes them to deviate from perfect focusing capabilities of an ideal metamaterial lens, subwavelength focusing is still achievable using a lossy negative index lens [38].

## 3. Simulation

**Figure 2:**
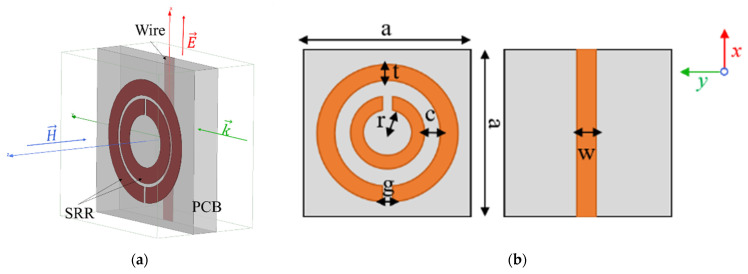
(**a**) HFSS unit cell model. (**b**) Schematic of the metamaterial unit cell showing both sides of the PCB.

A metamaterial can be designed by simulating an infinite array of metamaterial unit cells using periodic boundary conditions. Figure 2a shows an HFSS model of the proposed unit cell along with incident field polarization and direction of propagation. The *Perfect E* and *Perfect H* boundary conditions of HFSS were applied on the *y-z* and *x-y* boundaries, respectively, to mimic an infinite array of unit cells and ensure correct polarization of the incident wave. FR4 ($\epsilon_{r}=4.4$, tanδ=0.02) of thickness 1.6 mm was used as the substrate for the PCB. Copper of thickness 35 micron was used as the conducting material. Wave ports were assigned on the *z-x* boundary to excite the model with a plane wave and obtain the S-parameters of the metamaterial medium. Figure 2b shows the dimensions of the unit cell. The dimensional parameters for the proposed design at 3.45 GHz are as follows: r = 1.5 mm, c = g = 0.2 mm, t = w = 0.9 mm, and a = 9.3 mm. The distance between consecutive PCB layers (length of unit cell model along z direction) is 6.5 mm. The dimensions were adapted from previous works by Aydin et al., where they demonstrated negative refraction and left handed focusing by a metamaterial lens in the 3–4 GHz regime [39]. Although the principal objective of designing a negative index LHM lens is high spatial resolution, which can be achieved by improving the losses in the design, the primary purpose of this work is to demonstrate the viability of using an LHM lens for subwavelength defect detection. Hence, optimization of the performance of the unit cell by parameterizing its dimensions was left for future work.

### 3.1. Scattering Parameters

**Figure 3:**
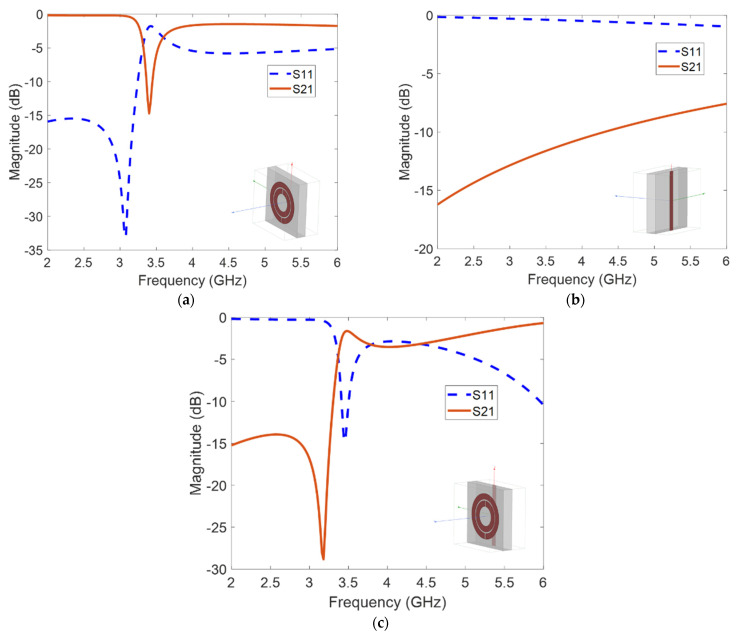
HFSS S-parameter results for (**a**) SRR-only medium, (**b**) wire-only medium, and (**c**) SRR and wire combined metamaterial medium. The respective HFSS models are shown in the insets of the figures.

Figure 3 shows the simulated S-parameters for three cases—an SRR-only medium, a wire-only medium, and a medium that combines both wires and SRRs. For the SRR-only medium, a dip in the transmission parameter *S*_21_ is observed around the resonant frequency of 3.45 GHz of the SRR (Figure 3a). This is due to the prohibition of propagating waves by the negative $\mu_{eff}$ of the medium. Figure 3b shows that the wire-only medium allows transmission (with less than 10 dB of insertion loss) above 5.5 GHz, which is the electric plasma frequency $\omega_{ep}$. Propagating waves below this frequency are prohibited due to the negative $\epsilon_{eff}$ of the wire medium. Figure 3c shows that after combining both the SRR and wire, a transmission band is observed around 3.45 GHz. Left-handed waves are allowed to propagate in the frequency region where both $\mu_{eff}$ and $\epsilon_{eff}$ are simultaneously negative.

### 3.2. Electromagnetic Parameter Retrieval

Extraction of the EM parameters from S-parameter data of the metamaterial design was done to verify left-handed nature of the transmission band. The procedure to determine the EM properties of the metamaterials is presented in Appendix A.

**Figure 4:**
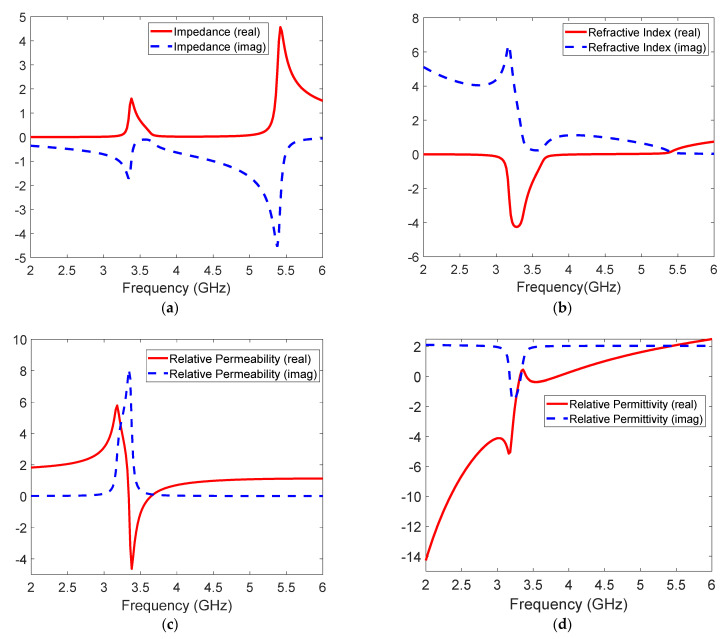
Extracted simulated EM parameters of the metamaterial design: (**a**) impedance, (**b**) refractive index, (**c**) permeability, and (**d**) permittivity.

The extracted material properties for the combined SRR and wire medium simulation results are shown in Figure 4. From the normalized impedance curve in Figure 4a, a resonance near the two plasma frequencies (3.45 GHz and 5.5 GHz) of the metamaterial medium is observed as expected. Figure 4b shows that the real part of the extracted refractive index is negative, thus verifying the left-handed transmission band in this frequency region. The value of real part of *n* at the resonant frequency of 3.45 GHz is −2.18. The real parts of the extracted $\mu_{eff}$ and $\epsilon_{eff}$ are also simultaneously negative in the frequency region, as expected (Figure 4c,d). It should be noted that above 5.5 GHz, both $\mu_{eff}$ and $\epsilon_{eff}$ are simultaneously positive, rendering the refractive index to be positive above this frequency.

## 4. Experiment

**Figure 5:**
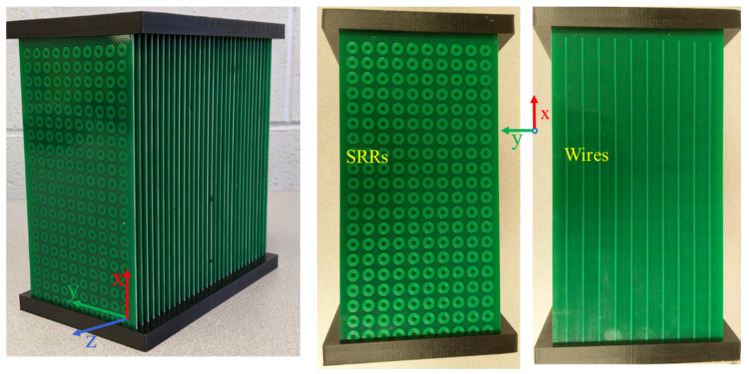
Fabricated metamaterial lens.

A metamaterial lens, consisting of $N_{x}=20$, $N_{y}=10$ and $N_{z}=31$ unit cells, was fabricated for experimental validation. Figure 5 presents the fabricated metamaterial lens. An amount of 20 × 10 unit cells in the *x-y* plane were printed in a single FR4 PCB of thickness 1.6 mm, and 31 such boards were stacked in the *z* direction at an interval of 6.5 mm. The magnetic and electric field vectors are polarized along the *z* and *x* axes, respectively, while the wave propagation vector is along the *y* axis. The thickness of the lens, *t*, in direction of propagation is 100 mm. Frequency sweep measurements were done to confirm the presence of left-handed transmission peak. A homodyne detection-based scheme was used to experimentally validate the negative refractive index and determine the subwavelength focal spot at the left-handed transmission peak frequency. NDE results for a set of dielectric test samples are presented in Section 4.2.3 to show the feasibility of using an LHM lens for the detection of subwavelength defects.

### 4.1. Transmission Characteristics

**Figure 6:**
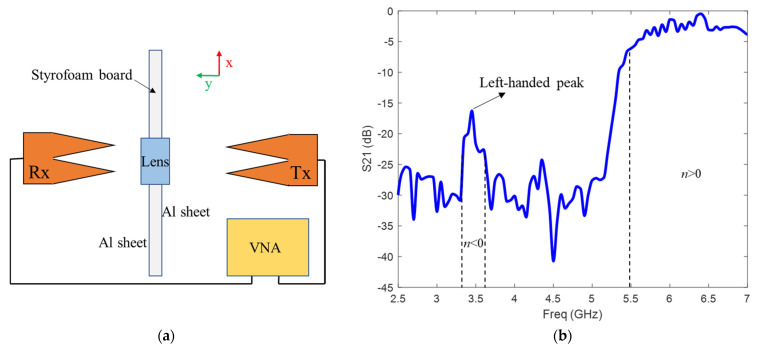
(**a**) Free space transmission experiment schematic. (**b**) Experimental transmission response of the metamaterial lens. The measurements were calibrated with respect to transmission in free space.

A large metallic screen (~10λ) with an aperture was used to obtain the transmission characteristics of the fabricated metamaterial lens. The metallic screen was implemented by attaching aluminum sheets to a Styrofoam board. An aperture of the size of the lens was cut in the middle of the board to allow for waves to pass through the lens only [40]. Wideband (675 MHz to 12 GHz) Vivaldi antennas were used as transmitter and receiver to illuminate the lens with a uniform plane wave. The antennas were placed 40 cm apart to ensure far-field measurements. The frequency sweep measurements were done using an Agilent EB070B vector network analyzer (VNA). Figure 6a shows the schematic of experimental set up. The measurements of *S*_21_ clearly indicate the presence of a left-handed transmission band with a peak transmission of −16 dB around 3.5 GHz (Figure 6b). The slight shift in frequency between the simulated and experimental results can be attributed to fabrication tolerances. Above 5.5 GHz, the metamaterial acts as a conventional medium having positive $\mu_{eff}$ and $\epsilon_{eff}$. Conventional right handed waves are allowed to propagate in this frequency regime, and hence, the transmission band is observed.

### 4.2. Left-Handed Characteristics

**Figure 7:**
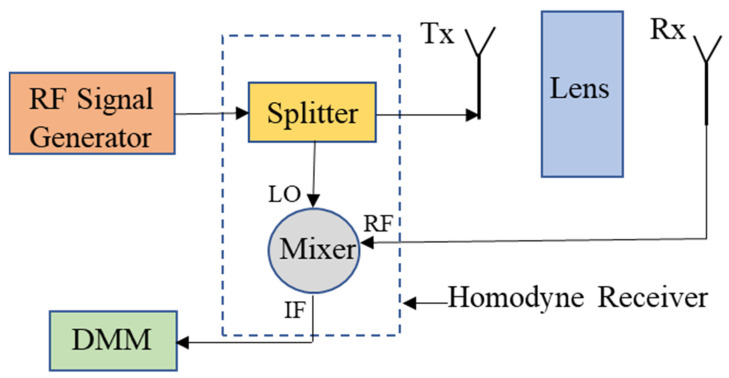
Schematic of homodyne architecture-based setup for single frequency measurements.

After experimentally confirming the left-handed transmission peak at 3.5 GHz, single frequency measurements using a homodyne receiver architecture were used to facilitate fast characterization of the metamaterial lens at 3.5 GHz. The schematic of the homodyne architecture is shown in Figure 7. The RF signal generator produces a continuous sinusoidal wave of frequency 3.5 GHz. The generated signal is passed through a splitter with one channel to the transmitting antenna and the other channel to the LO port of the mixer. The RF port of the mixer is connected to the receiver antenna. The DC signal produced at the IF of the mixer is read by a digital multimeter (DMM) and is proportional to the strength of the received signal. The high SNR associated with such synchronous detection allows the lens to be characterized in free space without the need for the windowing used in the frequency sweep measurements. Moreover, using homodyne detection circumnavigates the use of expensive RF instruments such as a VNA.

#### 4.2.1. Negative Refraction

**Figure 8:**
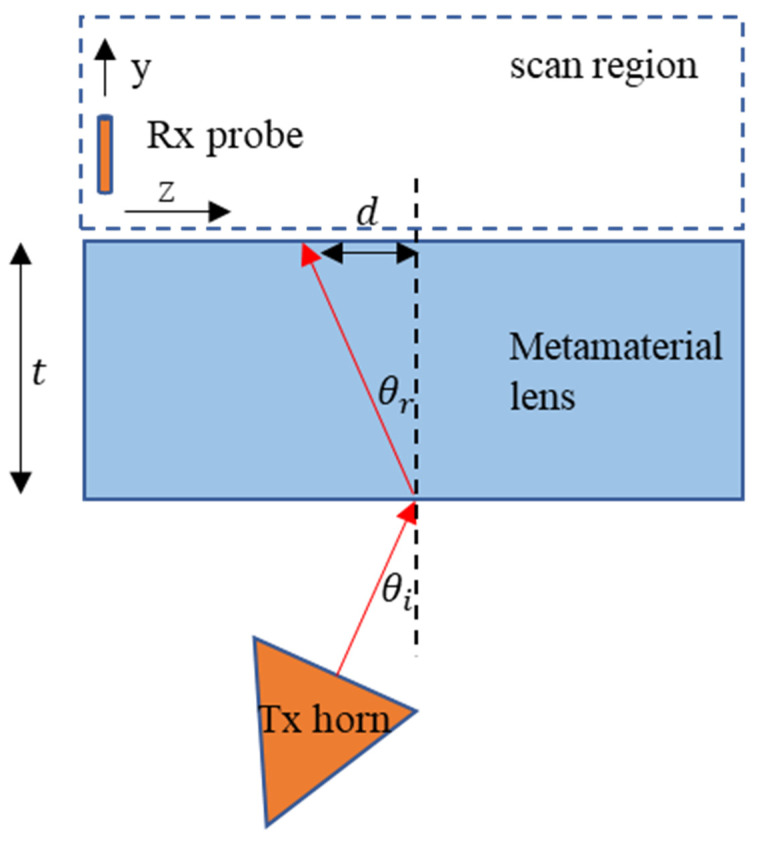
Negative refraction experiment schematic top view.

An imaging experiment was carried out to demonstrate negative refraction by the metamaterial lens and calculate its effective refractive index [33]. A standard gain horn antenna at 3.5 GHz was used as the transmitter, while a quarter wavelength monopole was used as the receiving probe. Outgoing spherical waves from the horn are incident at an angle $\theta_{i}$ at the first air-LHM interface. After undergoing negative refraction through the LHM of thickness *t*, the waves are shifted towards the side of the transmitter by a distance *d* at the second LHM–air interface. The angle of refraction can be determined by scanning the received signal amplitude can be calculated as $\theta_{r}=\tan^{-1}\left(d/t\right)$. The top view of the experimental setup is shown schematically in Figure 8.

**Figure 9:**
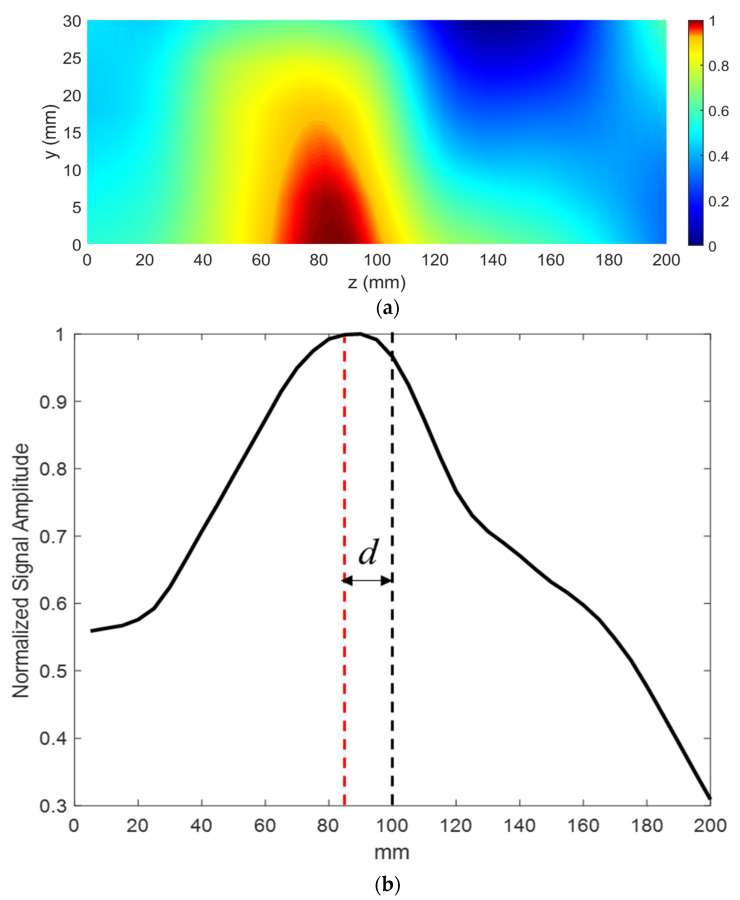
(**a**) Normalized received signal amplitude scan. The outgoing wave from the LHM has a beam profile centered at 85 mm. (**b**) Normalized line scan of the received signal amplitude at *y* = 0. The beam shift *d* is measured to be equal to 15 mm.

The receiving probe was mounted on a 2D scanner, moved in the *y-z* plane, and the received amplitude distribution was measured. A step size of 5 mm was used in both the *z* and *y* directions. The transmitting horn was placed at a distance of 12 cm (1.4λ) from the first air–LHM interface with an angle of incidence $\theta_{i}$ = 10°. The normalized measured amplitude distribution is shown in Figure 9a. The outgoing wave from the metamaterial lens has a beam profile centered towards the transmitting antenna. The angle of refraction, $\theta_{r}$, through the fabricated lens of thickness *t* = 100 mm and beam shift *d* = 15 mm is calculated to be 8.53°. The real part of effective refractive index is thereby computed using Snell’s law and is equal to −1.17 at 3.5 GHz.

#### 4.2.2. Subwavelength Focusing

**Figure 10:**
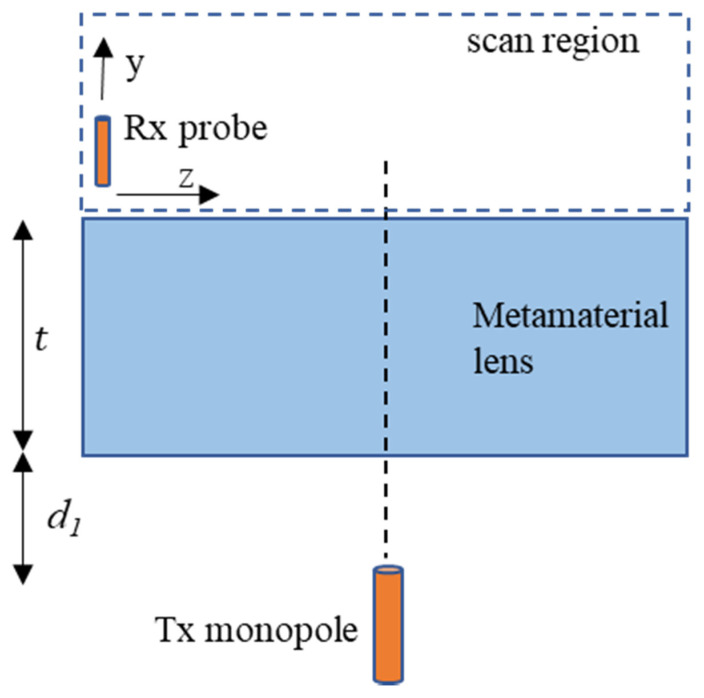
Subwavelength focusing experiment schematic top view.

The presence of negative refraction allows the possibility of using the fabricated metamaterial structure as a lens for subwavelength focusing. A monopole antenna produces an azimuthally symmetric field pattern, as does an ideal isotropic radiator. Therefore, a monopole with a resonant frequency of 3.5 GHz was used as the transmitter to demonstrate subwavelength focusing. Due to the negative refractive index of the metamaterial lens, diverging beams from the monopole antenna, placed at an appropriate distance, will be brought to focus outside the lens according to (5). Figure 10 presents the schematic top view of the experimental setup.

**Figure 11:**
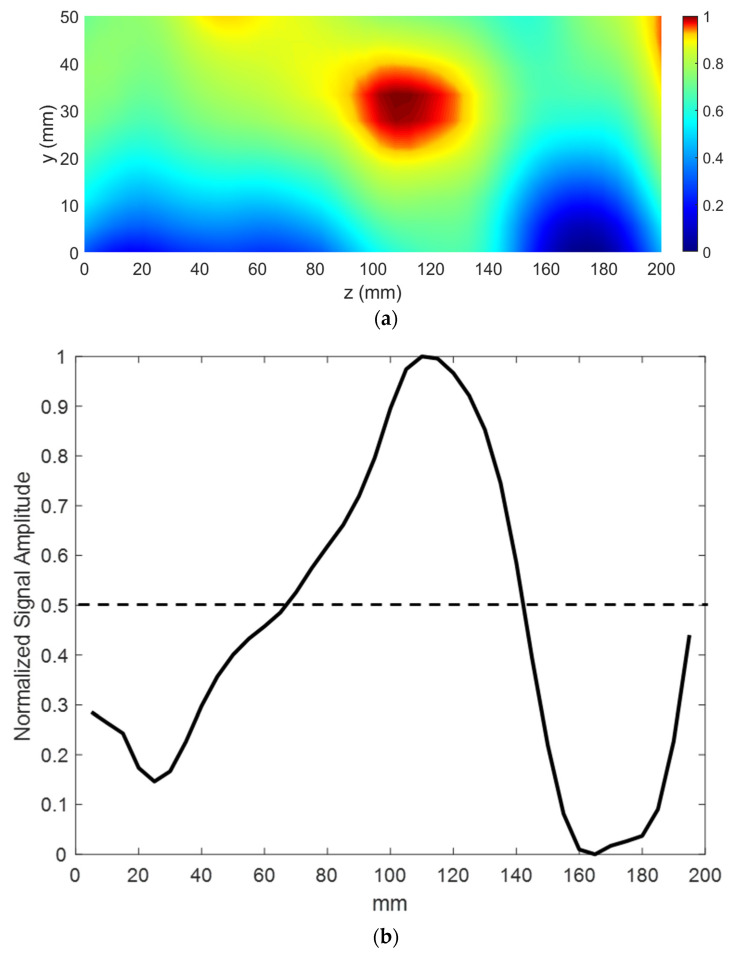
(**a**) Normalized received signal amplitude. A focal spot is observed at a distance of *d*_2_ = 30 mm from the lens. (**b**) Normalized line scan at *y* = 30 mm. The dashed lines indicate −3 dB point.

A quarter wavelength monopole was used as the probe for measuring the received signal. The receiving probe was mounted on a 2D scanner and moved on the *y-z* plane and the received amplitude distribution was measured. A step size of 5 mm was used in both the *z* and *y* directions. The transmitting monopole was placed at 60 mm (*d*_1_) from the air–LHM interface. Figure 11a shows normalized received signal amplitude. A focal point is observed at a distance 30 mm (*d*_2_) from the second LHM–air interface. The measured focal spot distance satisfies the relation in (5) for the fabricated lens of thickness 100 mm (*t*). Figure 11b shows the normalized line scan at the focal plane (*y* = 30 mm). The full width at half maxima (FWHM) for the focal spot is found to be 70 mm (0.82λ).

#### 4.2.3. Microwave NDE

**Figure 12:**
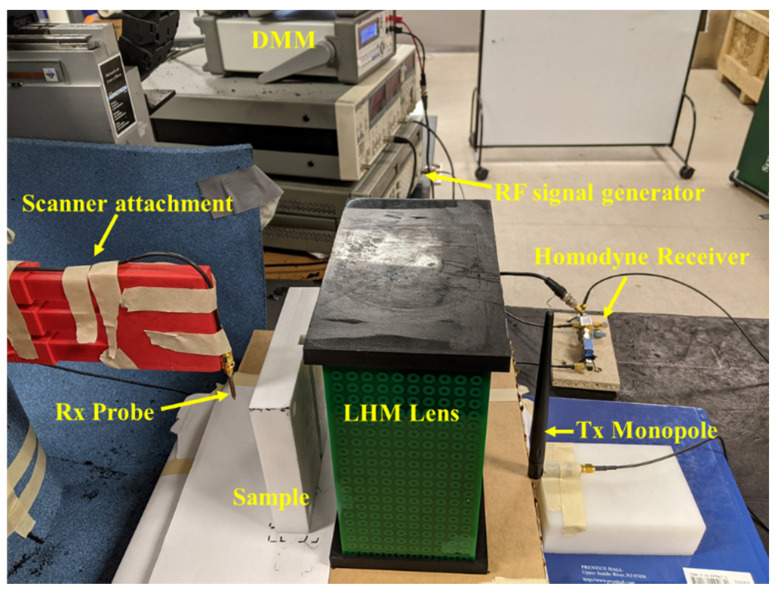
Proposed microwave NDE sensor. The sample is kept at the focal point of the LHM lens to allow for subwavelength defect detection.

**Figure 13:**
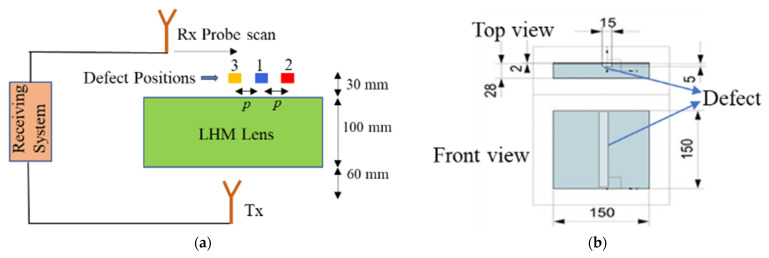
(**a**) NDE experiment schematic top view. (**b**) Sample under test schematic. All dimensions are given in mm.

**Figure 14:**
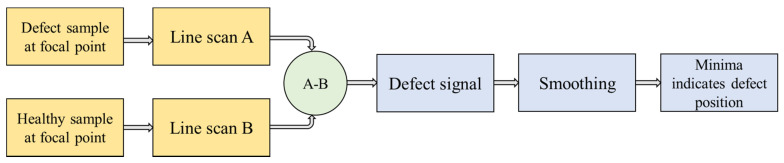
Calibration and defect detection flowchart.

Next, the capability of the LHM lens for detection of subwavelength defects with far-field microwave NDE data is demonstrated. The experimental setup is shown in Figure 12, and the schematic top view is shown in Figure 13a. Figure 13b shows the schematic of the sample under test. Teflon samples are used as the dielectrics for testing. A groove of size 15 mm (0.17λ) × 5 mm (0.06λ) is machined along the length of the sample. A similar Teflon sample with no machined groove is treated as the healthy sample. The samples are placed at the focal spot of the lens, and line scans are performed to obtain the scattered data. The contribution due to the defect is measured by the changes in the test signal relative to the baseline signal (found by measuring the healthy sample). The calibration and detection procedure is shown in Figure 14. The position of the defect can be determined by the minima in the line scans [26]. Three sets of measurements were taken at three positions of the sample. The distance *p* between consecutive defect positions was set to be 20 mm (0.23λ).

**Figure 15:**
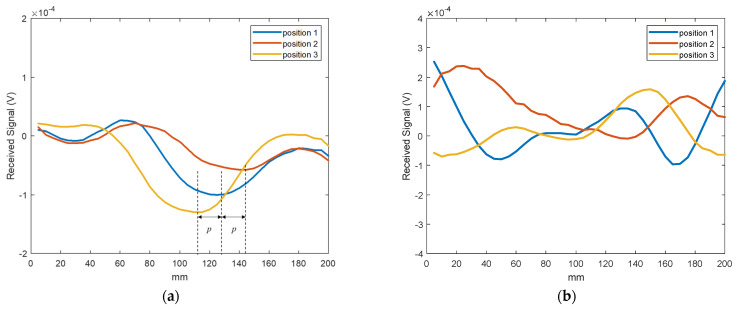
NDE line scan results. (**a**) Measurements using the LHM lens. The minimum of the plot gives the defect position. (**b**) Measurements without the LHM lens. Waves are not focused in free space. Subwavelength defects position cannot be determined.

Figure 15a shows the defect signal for the three measurements. The received line scans were fitted with smooth curves to obtain the minima. The minima in the three line scans are shifted with repositioning of the defect location, thus indicating the position of the subwavelength defect. This demonstrates the proposed system is sensitive to a 0.17λ × 0.06λ defect and can determine consecutive defect positions with a separation of 0.23λ. The NDE measurements were repeated without the LHM lens to illustrate that the subwavelength defects are not detectable without the lens. Figure 15b shows the resulting received line scan signals without the lens. Since waves from the monopole transmitter are not focused, the received signal strengths are dominated by edge effects and scattering from the background. Therefore, no minima, as in the case of the LHM lens, are observed. Hence, these initial results clearly demonstrate that subwavelength defects, which are undetectable in free space in the far field, can be detected using a properly designed metamaterial lens.

## 5. Discussion

This paper presents the design of a metamaterial lens and its application as a far-field microwave sensor. Numerical studies of the metamaterial unit cell design are presented. A fabricated metamaterial lens was used for experimental verification of the left-handed propagation characteristics of the lens. A homodyne detection setup was used with the LHM lens for NDE of subwavelength defects. Initial results demonstrate that a negative refractive index metamaterial lens can achieve resolution beyond the diffraction limit for far-field microwave NDE. The subwavelength resolution capability of a metamaterial lens is limited by the inherent losses associated with a fabricated LHM. Therefore, future work will involve the design of low loss LHMs to mitigate this issue. Moreover, work is in progress to design active metamaterials that can provide tunability that is lacking in passive metamaterial lens designs such as the one reported in this study. Finally, more extensive testing of experimental imaging of subwavelength defects is also underway to demonstrate the full potential of LHMs.

## Data Availability

Not applicable.

## Appendix A

Estimation of permittivity and permeability of an engineered material from its S-parameters is a well-established method, first proposed in the original work of Nicolson and Ross [41] and Weir [42]. Using this approach, an inhomogeneous metamaterial structure, assumed to be a homogenous medium under the effective medium theory, can be characterized by a refractive index *n* and normalized impedance z [43,44].

The normalized impedance z of a metamaterial unit cell is related to its S parameters by the equation

(A1) $$z=\pm \sqrt{\frac{{\left(1+S_{11}\right)}^{2}-S_{21}^{2}}{{\left(1+S_{11}\right)}^{2}+S_{21}^{2}}}$$

Since the metamaterial is a passive device, the reflected power cannot exceed the incident power. Therefore, the real part of z is positive, which in turn resolves the sign ambiguity in (A1).

The refractive index of a metamaterial unit cell of dimension *d* is related to the S-parameters by the following equation

(A2) $$e^{jnk_{0}d}=\frac{S_{21}}{1-S_{11}\frac{z-1}{z+1}}$$

The value of refractive index *n* can be evaluated from (A2) as

(A3) $$n=\frac{1}{k_{0}d}\left[\left\{Re\left(\ln\left(e^{jnk_{0}d}\right)\right)+2m\pi \right\}-j\left\{Im\left(\ln\left(e^{jnk_{0}d}\right)\right)\right\}\right]$$

where *Re*(.) and *Im*(.) are the real and imaginary operators, respectively, *k*_0_ is the free space wavenumber, and *m* is an integer. The ambiguity in the branch selection of the multi-valued complex logarithmic function in (A3) can be resolved by choosing correct integer value *m*, which is dependent on the electrical length of the unit cell. Due to the small electrical length of the proposed unit cell design (9.3 mm) compared to the homogenized wavelength (38 mm), the fundamental branch (*m* = 0) is chosen for calculation of material parameters from the simulation model [45].

The effective permittivity and permeability of the metamaterial are computed from *z* and *n* using the following relations

(A4) $$\mu_{eff}=nz;\;\epsilon_{eff}=n/z$$
